# Acute loss of the hepatic endo-lysosomal system *in vivo* causes compensatory changes in iron homeostasis

**DOI:** 10.1038/s41598-017-02898-4

**Published:** 2017-06-22

**Authors:** Christoph Metzendorf, Anja Zeigerer, Sarah Seifert, Richard Sparla, Bahar Najafi, François Canonne-Hergaux, Marino Zerial, Martina U. Muckenthaler

**Affiliations:** 1 Department of Pediatric Hematology, Oncology and Immunology University of Heidelberg, Heidelberg University Clinic, INF 350, 69120 Heidelberg, Germany; 2 Molecular Medicine Partnership Unit, 69120 Heidelberg, Germany; 3 Max Planck Institute of Molecular Cell Biology and Genetics, 01307 Dresden, Germany; 4 German Center for Diabetes Research (DZD), 85764 Neuherberg, Germany; 5 Institute for Diabetes and Cancer, Helmholtz Center for Environmental Health, 85764 Neuherberg, Germany; 6 IRSD, Université de Toulouse, INSERM UMR1220, INRA, ENVT, UPS, Toulouse, France; 7 Heidelberg University Biochemistry Center (BZH), INF 327, 69120 Heidelberg, Germany; 8 Institute for Diabetes and Cancer, Helmholtz Center for Environmental Health, 85764 Neuherberg, Germany

## Abstract

Liver cells communicate with the extracellular environment to take up nutrients via endocytosis. Iron uptake is essential for metabolic activities and cell homeostasis. Here, we investigated the role of the endocytic system for maintaining iron homeostasis. We specifically depleted the small GTPase Rab5 in the mouse liver, causing a transient loss of the entire endo-lysosomal system. Strikingly, endosome depletion led to a fast reduction of hepatic iron levels, which was preceded by an increased abundance of the iron exporter ferroportin. Compensatory changes in livers of Rab5-depleted mice include increased expression of transferrin receptor 1 as well as reduced expression of the iron-regulatory hormone hepcidin. Serum iron indices (serum iron, free iron binding capacity and total iron binding capacity) in Rab5-KD mice were increased, consistent with an elevated splenic and hepatic iron export. Our data emphasize the critical importance of the endosomal compartments in hepatocytes to maintain hepatic and systemic iron homeostasis *in vivo*. The short time period (between day four and five) upon which these changes occur underscore the fast dynamics of the liver iron pool.

## Introduction

Iron is an essential micronutrient. Due to its ability to undergo redox-reactions it serves as a cofactor for many enzymes. However, excess free iron causes the formation of toxic reactive oxygen species that damage proteins, lipids and nucleic acids. Hence, iron-balance must be accurately maintained at the cellular and organismal level (reviewed among others in refs 1–3).

On the organismal level, the liver detoxifies and stores iron and plays a central role in maintaining systemic iron homeostasis via the expression of the peptide hormone hepcidin. Hepcidin expression is regulated in response to iron levels involving proteins mutated in patients with Hereditary Hemochromatosis (HFE, TFR2 and HJV) and the BMP/SMAD1/5/8 signaling pathway. Additionally, inflammation results in increased expression of hepcidin through IL6/IL6R and JAK/STAT3 as well as ActivinB/SMAD1/5/8 signaling^4–13^. Hepcidin is secreted by the liver and binds to its receptor, the iron exporter ferroportin (IREG1, FPN1, SLC40A1). Ferroportin is mainly expressed in duodenal enterocytes, hepatocytes and reticuloendothelial macrophages, and its abundance on the cell surface regulates iron export into plasma. Hepcidin binding triggers ferroportin endocytosis and ubiquitin-mediated proteolytic degradation^14–16^. Thus, elevated hepcidin levels in response to high systemic iron availability decrease iron uptake from the diet and iron release from iron stores. Inadequate expression of hepcidin causes frequent iron related disorders, such as Hereditary Hemochromatosis, iron refractory iron deficiency anemia or the anemia of inflammation^17^.

Iron released into the bloodstream via ferroportin is oxidized by ferroxidases and binds to the plasma protein transferrin. Holo-transferrin interacts with the transferrin receptor (TFR1) and is taken up by cells via clathrin-mediated endocytosis^18^. In acidified endosomes iron is released from transferrin, reduced, and transported across the membrane by divalent metal transporter (DMT)1^19, 20^. Non-transferrin-bound iron (NTBI), detectable in the plasma under conditions of high transferrin saturation, is efficiently taken up by the liver via zinc transporter protein ZIP14 (SLC39A14)^21, 22^. ZIP14 localizes to the cytoplasmic membrane and endosomes^23^ and endocytosis is required for efficient NTBI uptake^24^. Within hepatocytes, iron is stored in the iron storage protein ferritin. Iron mobilization from ferritin requires its degradation via the lysosomal compartment^25^ and transfer of iron from early endosomes to mitochondria via membrane contact sites (MCS) occurs at least in endothelial cells^26^ and reticulocytes^27^. Thus, several critical steps involved in maintaining cellular iron homeostasis require endosomal compartments. So far, the direct requirement of the endosomal system for maintaining iron homeostasis has not been established.

Rab5 is essential for endosome biogenesis and the maintenance of the endo-lysosomal system^28, 29^. Previous work demonstrated that RNAi of *Rab5* caused a reduction of early and late endosomes and lysosomes at day four and five post RNAi injection, establishing Rab5 as the master regulator of endosomal biogenesis^29^. The same experimental strategy was applied here to investigate the consequences of the loss of the endo-lysosomal system on hepatocellular and systemic iron homeostasis. Strikingly, the short time span (24–48 hrs) during which the endolysosomal system is significantly ablated in this model^29^ was sufficient to significantly reduce liver iron levels and cause compensatory responses, exemplifying the highly dynamic nature of the liver-iron pool.

## Results and Discussion

### Endosome depletion in hepatocytes causes reduced hepatic iron levels

To investigate the role of the endo-lysosomal system for maintaining iron homeostasis, we silenced the three isoforms of *Rab5* (*Rab5a*, *b*, *c*)^28, 29^. This was achieved by injection of lipid nanoparticles (LNPs) containing small interfering RNAs through the tail vein^29^. We previously demonstrated that RNAi of all three *Rab5* isoforms caused a 50% decrease of Rab5 protein levels three days after a single siRNA injection, without affecting endosome numbers. Early and late endosomes and lysosomes were however reduced dramatically at day four and five post injection^29^. At day 10 post-injection, the endo-lysosomal system was restored to its normal state^29, 30^.

Here we confirm that all three *Rab5* isoforms were successfully depleted in the liver three, four and five days post siRNA injection (Supplementary Figure S1A–C), while no changes were observed in spleen (Supplementary Figure S1D), indicating high specificity of this RNAi approach for liver/hepatocytes. Previous work further demonstrated that the transient depletion of the endo-lysosomal system does not cause inflammatory or liver-damaging effects as serum IL-1b, IL6, TNFα, AST (aspartate amino-transferase activity) and ALT (alanine amino-transferase activity) levels remained unaffected and lethality of the mice was not observed^29, 30^. Consistently, we show that liver *IL6*, *Saa2* and *F4/80* as well as spleen *IL6* mRNA levels remained unchanged (Supplementary Figure S1E–H). Thus, inflammatory signals previously reported to control iron homeostasis^1, 31, 32^ are unlikely to influence cellular and systemic iron homeostasis in this model.

**Figure 1:**
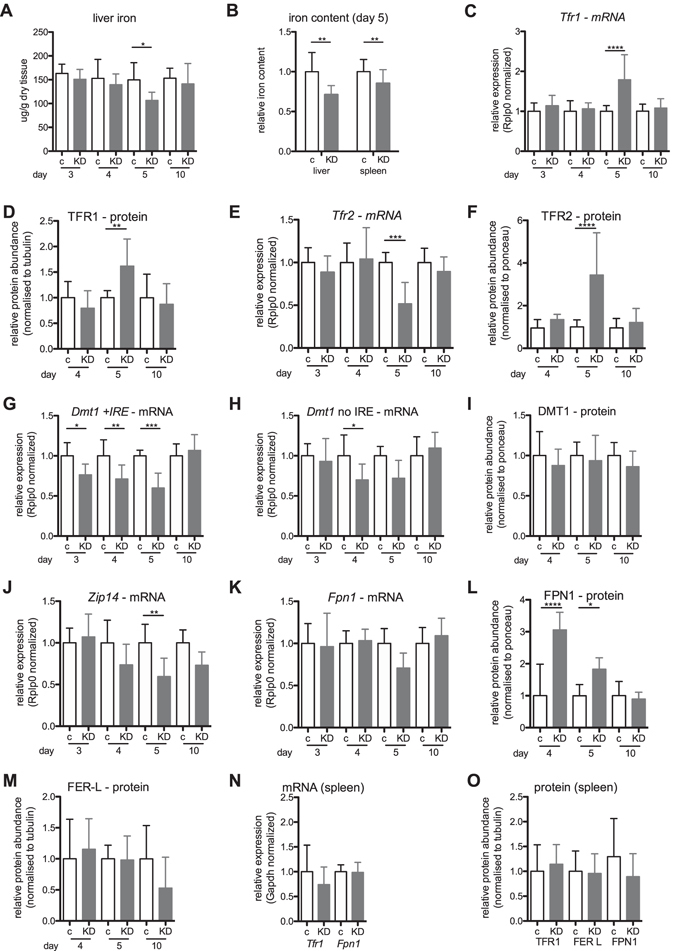
Iron-related parameters in liver and spleen of Rab5-KD (KD) and control mice (c). (**A**) Non-heme iron concentrations in liver. (**B**) Relative iron concentrations in liver and spleen. Relative mRNA (**C**, **E**, **G**, **H**, **J**, **K** and **N**) and protein (**D**, **F**, **I**, **L**, **M** and **O**) levels in liver (**C**–**M**) and spleen (**N** and **O**) of transferrin receptor (*Tfr1*/TFR1; **C**, **D**, **N** and **O**), Tfr2/TFR2 (**E** and **F**), divalent metal transporter 1 (*Dmt1*/DMT1; **G**, **H** and **I**), *Zip14* (**J**) ferroportin (*Fpn1*/FPN1; **K**, **L**, **N** and **O**) and ferritin light chain (FER-L; **M)** as determined by qPCR and semi-quantitative western blot analysis, respectively. Normalization was carried out as indicated; proteins detected in membrane fractions from liver samples were normalized to ponceau stained membrane. “c” = control, “KD” = Rab5-KD; day 3–5 refer to days post-RNAi. See Supplementary Figures S2–S4 for representative blots. One-way ANOVA with Bonferroni correction for comparison of selected pairs for all panels except (**B**) two-way ANOVA. *P < = 0.05, **P < = 0.01, ***P < = 0.005 and ****P < = 0.001.

To study the effect of Rab5 loss on liver iron homeostasis, we compared liver iron levels in control and Rab5-depleted mice. Strikingly, five days post RNAi-treatment we detected a 29% decrease of liver iron levels (Fig. 1A). This is surprising considering the relatively short time period during which the endo-lysosomal system is depleted (approximately 24–48 hrs, between days four and five post-LNP injection^29^). In addition, splenic iron levels were mildly reduced as indicated by two-way ANOVA analysis (Fig. 1B). To identify putative causes for the decrease of tissue iron content, we next analyzed the expression levels of mRNAs and proteins that play critical roles in maintaining cellular iron homeostasis. We show that mRNA and protein expression of transferrin receptor 1 (*Tfr1*), and protein levels of TFR2 are significantly increased in livers of Rab5-KD mice at day five post siRNA treatment; however, at the same time point *Tfr2* mRNA levels were significantly decreased (Fig. 1C–F). mRNA levels of both, the iron responsive element (IRE)-containing isoform of *Dmt1* as well as its splice variant without the IRE were decreased in Rab5-depleted livers (Fig. 1G and H), while changes on the protein level were not observed (Fig. 1I). In addition, ZIP14 mRNA levels were reduced (Fig. 1J; determination of ZIP14 protein levels was not possible (data not shown)). By contrast, mRNA levels of the iron export protein ferroportin remained unaltered (Fig. 1K), while its protein level was significantly increased on days four and five post siRNA treatment (Fig. 1L). Expression of the iron storage protein ferritin was not significantly changed in the liver (Fig. 1M).

**Figure 2:**
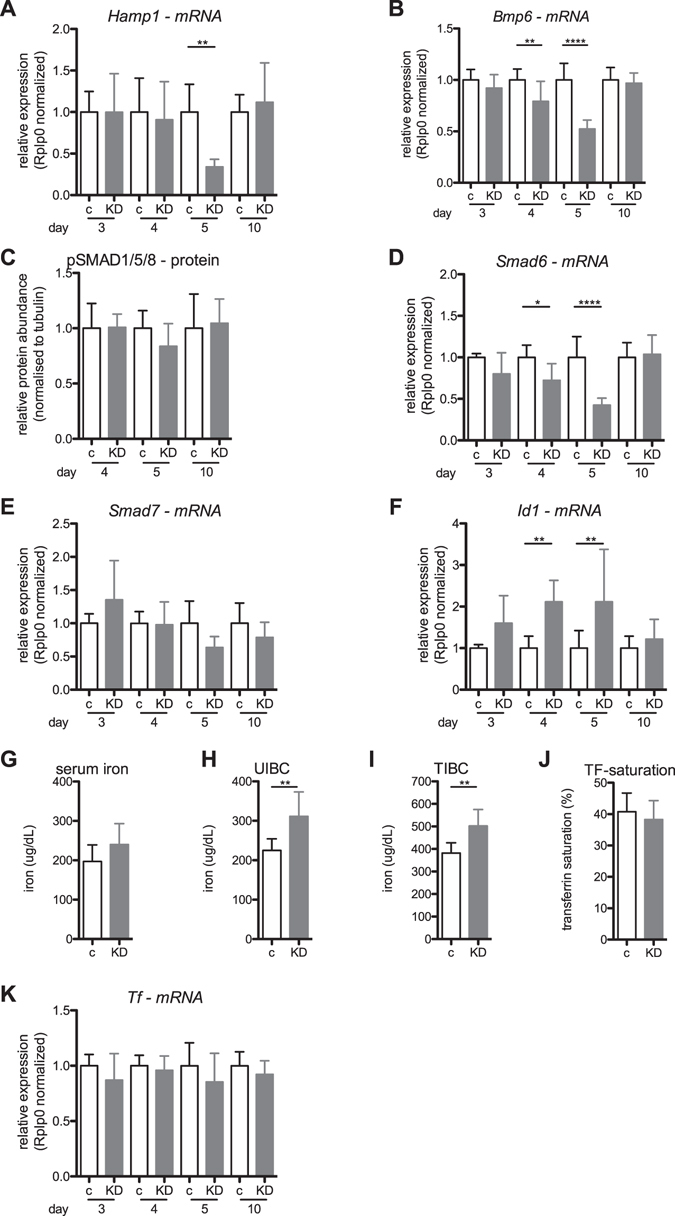
Systemic iron-related parameters in Rab5-KD (KD) and control mice (c). Relative mRNA expression of *hepcidin* (*Hamp1*; **A**), *Bmp6* (**B**), *Smad6* (**D**), *Smad7* (**E**), *Id1* (**F**) and *transferrin* (*Tf*, **K**) in liver, as determined by qPCR. (**C**) Relative abundance of phosphorylated SMAD1/5/8 as determined by semi-quantitative western blotting from tissue lysates normalized to tubulin expression (see Supplementary Figure S3 for representative blot). (**G**) Serum iron concentration, (**H**) unsaturated iron binding capacity (UIBC), (**I**) total iron binding capacity (TIBC) and (**J**) transferrin saturation in serum of control and Rab5-KD mice. “c” = control, “KD” = Rab5-KD; day 3–5 refer to days post-RNAi. (**A**, **B** and **D**–**G**) One-way ANOVA with Bonferroni correction for comparison of selected pairs. (**C**) Student´s t-test. *P < = 0.05, **P < = 0.01, ***P < = 0.005 and ****P < = 0.001.

In contrast to the liver, *Tfr1* and *Fpn1* mRNA and protein levels and ferritin protein levels remained unaltered in the spleen on day five post siRNA treatment (Fig. 1N and O), consistent with a liver-specific effect of Rab5-KD on iron homeostasis. Reduced splenic iron levels may be explained by the decreased hepatic hepcidin expression (Fig. 2A and next section).

Post-transcriptional control of iron-related genes via the iron regulatory protein (IRP)/IRE system is critical to maintain cellular iron homeostasis. Under iron-deficient conditions, IRPs bind to IREs located in the 3′ untranslated region of the *Tfr1* mRNA, resulting in its protection from RNase-mediated degradation^33^. Hence, increased *Tfr1* mRNA and protein levels in the livers of Rab5-KD mice are likely a compensational response to low cellular iron levels (Fig. 1A,C and D). Additionally, TFR1 protein may accumulate in Rab5-KD cells due to the depletion of the endo-lysosomal system^29^, which is required for TFR1 endocytosis, recycling and degradation. Despite its increased expression in Rab5-KD mice, TFR1 seems to be unable to increase cellular iron levels in the liver to compensate for iron losses caused by increased ferroportin protein expression. Compared to TFR1, TFR2 binds to holo-transferrin with much reduced affinity^34^ and, thus, may not play a significant role in reverting hepatic iron deficiency. TFR2 is rather involved in iron sensing and the regulation of hepcidin expression^35, 36^.

In conditions of liver iron deficiency, ferritin is (1) translationally repressed by IRP binding to its 5′UTR IRE and (2) degraded in lysosomes to release iron^25^ and thus would be expected to show reduced expression. However, ferritin levels are not significantly different between Rab5-KD and control mice (Fig. 1M), suggesting impairment of these processes in Rab5-KD mice.

The finding that ferroportin mRNA levels were not changed and protein abundance was significantly increased in Rab5-KD livers at day four and five post RNAi treatment (Fig. 1K and L) may be explained by an impairment of ferroportin degradation. Ferroportin is degraded in lysosomes^37^, and thus the loss of the endo-lysosomal system in Rab5-KD mice is expected to cause reduced clearance of the protein from the cell surface as well as reduced protein degradation. Likewise, other proteins with a localization in plasma or endosomal membranes, such as TFR2 and DMT-1 also show increased protein levels in relation to their mRNA levels (Fig. 1E–I). This finding is consistent with comparative micro-array and proteomics analysis of Rab5-KD mouse livers, which demonstrates that numerous transmembrane proteins are more abundant than their mRNA expression would suggest^30^. More specifically, due to the strong reduction of the endo-lysosomal system upon Rab5-KD, canonical endocytic transport processes, such as receptor-mediated clathrin coated LDL internalization and degradation are dramatically reduced^29^, resulting in protein amounts of the LDL-receptor that are increased despite unaltered mRNA expression. Because the recycling and secretory pathways are almost unaffected^29^ this provides the cell with a continuous supply of transmembrane proteins^29, 30^.

Ferroportin protein levels are already increased at day four after *Rab5*-RNAi, contrasting the responses of other membrane proteins and the hepatic iron level, which are altered on day five. This suggests that increased iron release from hepatocytes may be the primary reason for the decrease of liver iron levels that cannot be compensated for by elevated iron uptake through holo-transferrin/TFR1, DMT1 or ZIP14. Impaired iron uptake in hepatocytes is explained by compromised endocytosis in Rab5-KD cells^29^ that is required for both, the uptake of transferrin bound iron and non-transferrin bound iron^18, 24^. Additionally, reduced hepcidin expression observed in Rab5-KD livers (Fig. 2A) will contribute to the stabilization of ferroportin protein on the cell membrane of hepatocytes^15, 16, 38^. These findings are consistent with data obtained by a systems biology approach which predicted that the liver iron pool is highly dynamic and is maintained through a high iron uptake-rate in addition to iron storage in ferritin^39^. Taken together our data suggest that depletion of the endo-lysosomal system causes elevated iron export via ferroportin and reduced hepatic iron levels, which cannot be compensated by an increased rate of iron uptake.

### Alterations in systemic iron homeostasis induced by endosome depletion

We next analyzed whether the depletion of endosomes alters hepcidin levels and the pathways regulating its expression. Consistent with decreased hepatic iron concentrations, we observed significantly reduced *Bmp6* and *hepcidin* mRNA levels as well as reduced SMAD1/5/8 phosphorylation at day five post-RNAi (Fig. 2A–C), whereby reduced *Bmp6* mRNA expression preceded the hepcidin response by one day (Fig. 2A and B). These findings suggest that the iron sensing process adequately responds to decreased hepatic iron levels in endosome-depleted livers, despite elevated levels of the hepcidin activator TFR2 (Fig. 1F). While *Smad6* and *Smad7* mRNA levels were decreased consistent with attenuated SMAD1/5/8 phosphorylation and decreased *hepcidin* expression, *Id1*, an additional target gene of BMP6/SMAD signaling, which is frequently co-regulated with hepcidin^9^, did not show the expected decrease in mRNA expression (Fig. 2D–F). Interestingly, *Id1*, a regulator of energy metabolism^40^ was significantly induced in Rab5-KD livers on day four and five (Fig. 2F). This may be explained by an increased glycolytic flux in Rab5-KD livers^30^ and a function of ID1 in promoting a metabolic shift towards aerobic glycolysis^41^.

Our molecular analysis demonstrates elevated ferroportin protein levels in the liver of Rab5-KD mice. As a consequence, we would expect an increased iron export rate, which may be reflected by elevated serum iron levels. Indeed, serum analysis shows a mild increase in serum iron levels (Fig. 2G), albeit only statistically significant when data of independent experiments are normalized to the respective control group (Supplementary Figure S5D). In addition, unsaturated iron binding activity and total iron binding capacity were increased to a similar degree as serum iron levels, resulting in a transferrin saturation that was not significantly different in Rab5-KD mice compared to control mice (Fig. 2H–J).

Based on previous observations that levels of liver-secreted proteins such as factor VII, albumin and HDL are not altered in the Rab5-KD model^29^, we expect that hepcidin secretion is intact. Furthermore, an increase in the iron binding capacity in Rab5-KD mice is only possible if secretion of the plasma protein transferrin, which is predominantly synthesized in the liver, is adequate. We speculate that increased transferrin levels may be a result of a decreased uptake/degradation rate by the liver, because transferrin transcript levels are not increased (Fig. 2K).

Taken together, this study shows that Rab5 and the endo-lysosomal system are required for iron homeostasis. As the depletion of the endo-lysosomal system occurs within a 24–48 hour period, between day three and five post treatment (maximal on day five^29^), it was surprising to observe such a rapid drop in liver iron levels. Reduced hepatic iron levels in Rab5 depleted mouse livers are very likely initiated by increased iron export from the liver through ferroportin. Because all iron uptake processes require the endo-lysosomal system, compensation by increased iron uptake via TFR1/DMT1/ZIP14 is not possiblel^29^. In addition, iron mobilization from ferritin, which requires lysosomal degradation, is likely impaired. Thus, compensatory responses to counteract hepatic iron deficiency are inactive in the absence of the endosomal system, causing a negative feedback that worsens hepatocyte iron depletion.

## Materials and Methods

### Animals and LNP injections

Animal studies were approved and conducted in accordance with German animal welfare legislation and in specific pathogen-free conditions in the animal facility of the MPI-CBG, Dresden, Germany. Protocols were approved by the Institutional Animal Welfare Officer (Tierschutzbeauftragter), and necessary licenses were obtained from the regional Ethical Commission for Animal Experimentation of Dresden, Germany (Tierversuchskommission, Landesdirektion Dresden). LNP injections and Rab5 silencing in mice were performed as described before^29^.

### Tissue and serum iron quantification

Tissue iron was measured in dried tissue samples using acid extraction and bathophenantroline as established by^42^ and modified by^43^. Because absolute iron levels between control animals of different experiments varied when samples of each experiment were processed separately on different days, we normalized tissue iron levels by dividing each value by the average iron level of the control group of the respective experiment to determine relative iron levels to compare liver and spleen iron levels (Supplementary Figure S5A–D).

Serum iron was measured using the bathophenantroline based method of the SFBC kit 80008 (BIOLABO, France) adapted to 96-well manual format by scaling to 40 uL sample volumes^44^. Unsaturated iron binding capacity (UIBC) in serum was determined using the UIBC kit 97408 (BIOLABO, France) adapted to 96-well format by scaling to 20 uL sample volumes^44^.

### RNA extraction, cDNA synthesis and qPCR

Total RNA was purified from tissue samples using Trizol reagent (life technologies) according to the manufacturer’s protocol applying one additional ethanol-wash of the RNA prepared from liver to reduce high A _230 nm_. 2 μg total RNA were reverse transcribed using random hexamers and RevertAid (Fermentas). qPCR was performed on a StepOne thermocycler (Applied Biosystems) using the SYBR-green Master Mix (Applied Biosystems) and primers listed in Supplementary Table S1. Relative mRNA expression was calculated by the delta Ct method and normalized to the reference gene *Rplp0* ^29, 45^.

### Western blot analysis

SDS-PAGE and western blot analysis of total protein lysates were performed as previously described^46, 47^. DMT1 antiserum and affinity purified antibody were used as described in^48^.

Microsome/cytoplasm membrane extracts (membrane fraction) were obtained by homogenizing tissue samples in hypotonic buffer (10 mM Tris HCl, 2 mM MgCl2 and protease inhibitor mix (Roche) using a glass homogenizer. The supernatants obtained from centrifuging homogenates for 10 min at 4 °C and 1000 rpm were sonicated (Bandilin Sonoplus) for 15 min on ice before adding 250 mM sucrose and centrifuging at 1000 × g for 10 min at 4 °C to remove nuclei. The resulting supernatants were centrifuged at 12000 × g at 4 °C for 15 min to remove mitochondria. Microsomes and plasma membranes were obtained by centrifugation of the supernatants from the previous step at 105000 × g at 4 °C for 60 min.

To normalize sample data between different experiments and western blots we generated a standard sample that was applied in triplicate to SDS-PA gels where applicable. Western blots were imaged and analyzed using the Fusion-FX system (Vilber Lourmat) or ECL and x-ray film and BioRad QuantityOne or ImageJ software. Western blot signals were normalized by the signal of tubulin. As depletion of the endolysosomal pathway will result in the accumulation of many membrane proteins, signals obtained from membrane fractions were normalized to total protein levels as quantified by densitometry of the Ponceau signal per sample lane.

## Electronic supplementary material

Supplementary  Information
