# Supplementary material for: Acute loss of the hepatic endo-lysosomal system in vivo causes compensatory changes in iron homeostasis
Source: Sci Rep. 2017 Jun 22;7:4023. doi: 10.1038/s41598-017-02898-4 (PMC5481338; doi:10.1038/s41598-017-02898-4)
Supplement: Supplementary file 1 — Supplementary Information [file 41598_2017_2898_MOESM1_ESM.pdf]

Supplementary Information

Acute loss of the hepatic endo-lysosomal system *in vivo* causes compensatory changes in iron homeostasis

Christoph Metzendorf, Anja Zeigerer, Sarah Seifert, Richard Sparla, Bahar Najafi, François Canonne-Hergaux, Marino Zerial and Martina U. Muckenthaler

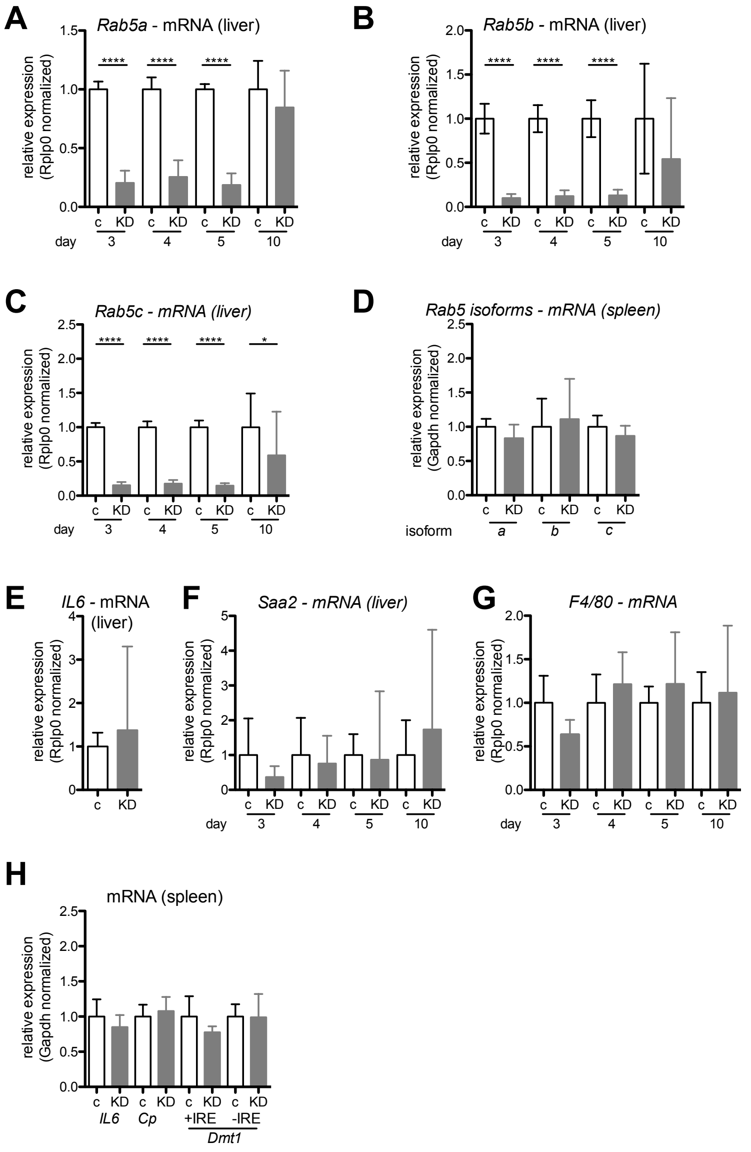

Supplementary Figure S1: Expression of *Rab5* isoforms *a*, *b* and *c* in control (c) and Rab5-KD (KD) mice in liver (days 3, 4, 5 and 10 post RNAi treatment, **A-C**) and spleen (day 5 post RNAi treatment, **D**). mRNA expression of inflammatory markers in liver (**E-G**) and spleen (*IL6*, **H**). Primers to analyze Rab5a-c were used according to[22], all other primers are listed in table S1. Data from 1-2 independent experiments, n=4 per treatment and experiment. C = control, KD = Rab5-KD; day 3-5 refer to days post-RNAi; panels A-D one-way ANOVA with Bonferroni correction (comparing selected pairs); panels C and D Student´s t-test. * P <= 0.05, ** P <= 0.01, *** P <= 0.005 and **** P <= 0.001.

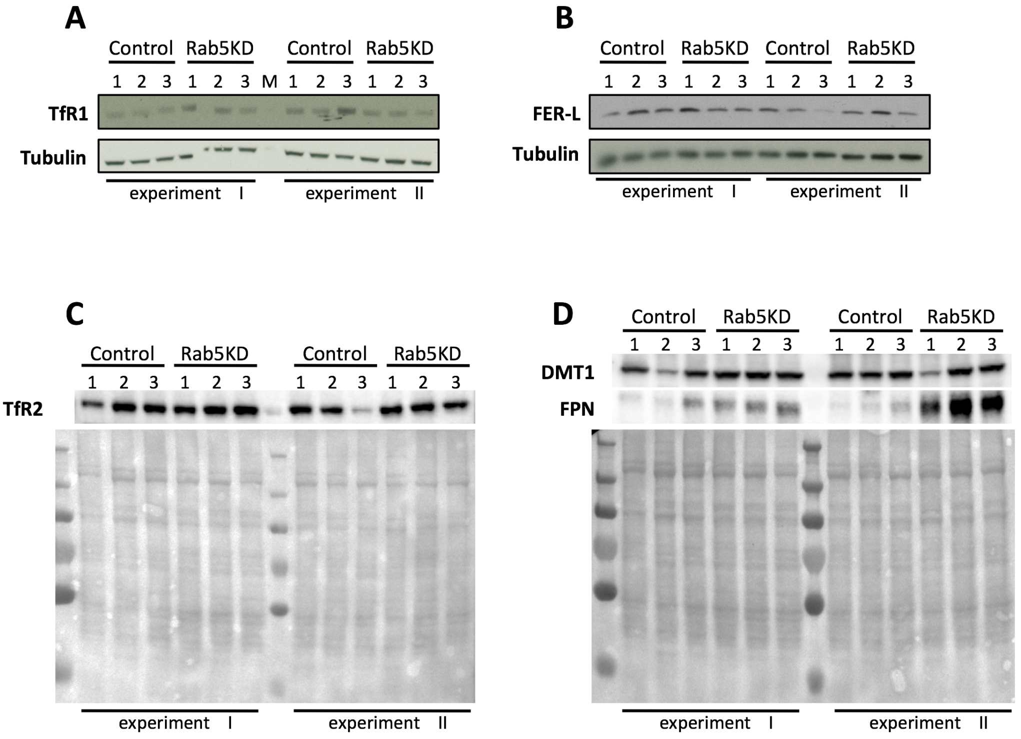

Supplementary Figure S2: Western blot analyses of iron proteins in livers of control and Rab5-KD mice from day 4 post RNAi-treatment using TFR1 (**A**), FER-L (**B**), TFR2 (**C**), DMT1 and FPN (**D**) antibodies. Whole liver lysates were used for TFR1 and FER-L and membrane fractions of liver lysates were prepared for TFR2, DMT1 and FPN analyses. For semi-quantitative analyses signals were normalized with tubulin signals or ponceau stained whole-lane signals for whole lysates and membrane fractions, respectively.

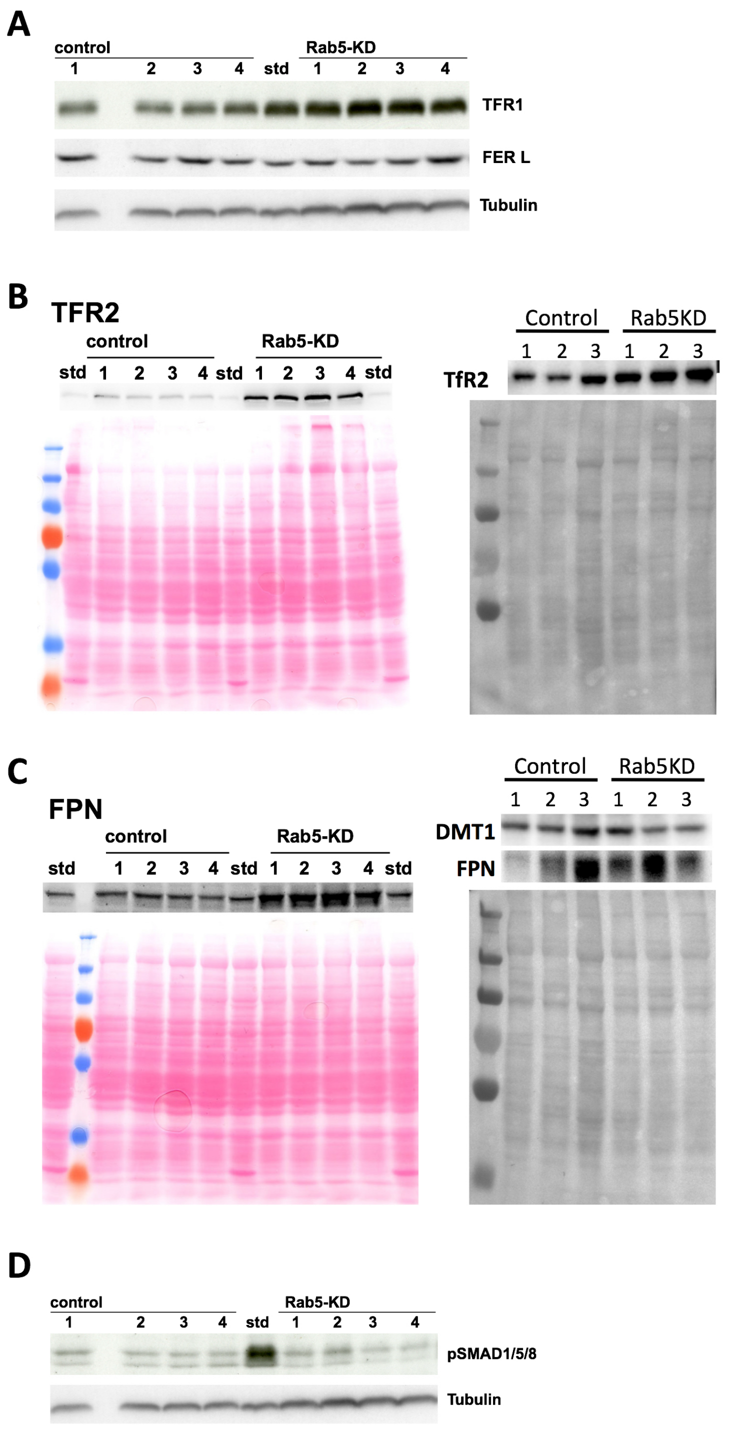

Supplementary Figure S3: Representative western blots of whole liver homogenates (**A** and **D**) and of membrane enriched samples (**B** and **C**) from control and Rab5-KD mice. To allow for the relative quantification of data acquired from different blotting membranes a standard sample (std) was used in triplicate for each western blot where applicable. For semi-quantitative analyses signals were normalized with tubulin signals or ponceau stained whole-lane signals for whole lysates and membrane fractions, respectively.

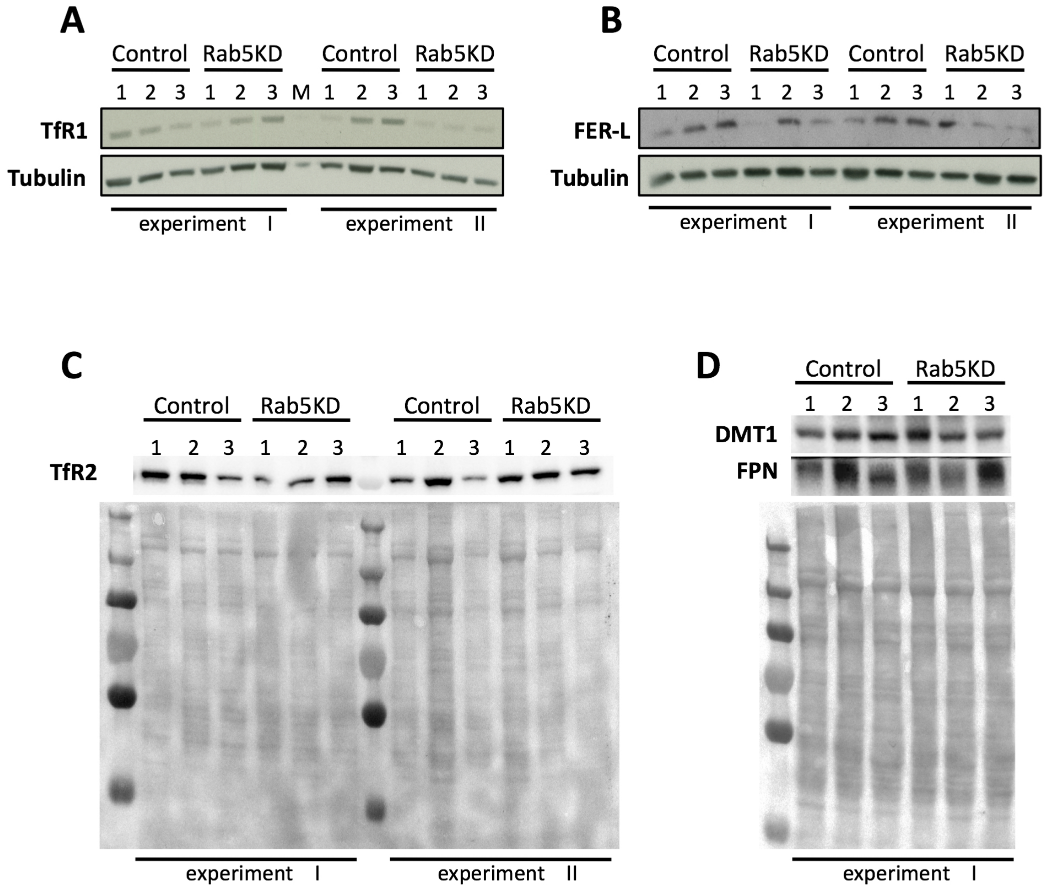

Supplementary Figure S4: Western blot analyses of iron-related proteins in livers of control (c) and Rab5-KD mice from day 10 post RNAi-treatment using TFR1 (**A**), FER-L (**B**), TFR2 (**C**), DMT1 and FPN (**D**) antibodies. Whole liver lysates were used for TFR1 and FER-L analysis and membrane fractions of liver lysates were used for TFR2, DMT1 and FPN analysis. For semi-quantitative analysis signals were normalized with tubulin signals or ponceau stained whole-lane signals for whole lysates and membrane fractions, respectively.

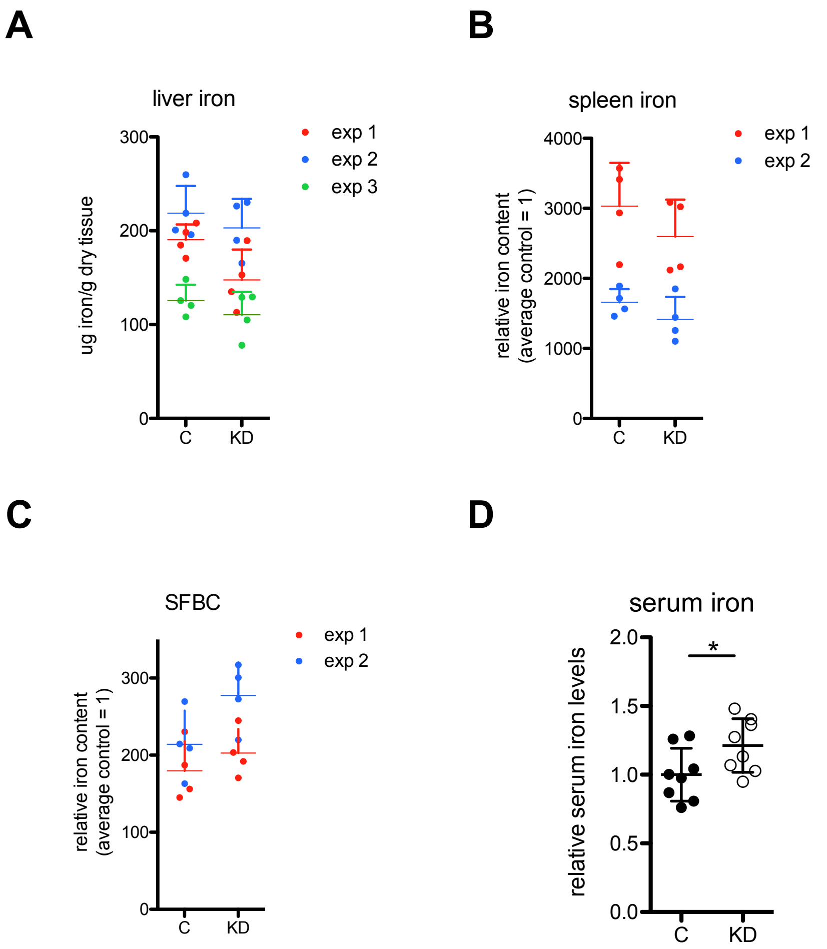

Supplementary Figure S5: (**A**) Liver iron (**B**) spleen iron and (**C**) serum iron (SFBC) concentrations are color-coded for each individual experiment (exp 1-3). A consistent trend for lower iron concentrations in liver and spleen and for higher iron concentrations in the serum of Rab5-KD mice was observed. However, absolute iron concentrations in control animals differed between different experiments. Therefore, the relative iron content was calculated within each experimental group by dividing the iron content of each sample of the same experiment by the average of the iron content of the samples of the corresponding control group. (**D**) Normalization per experiment resulted in a significant difference in serum iron levels between the Rab5-KD and control mice. C = control, KD = Rab5-KD.

Supplementary Table S1: Primers used for qPCR

primer name	sequence 5´ -> 3´
Bmp6 F	CCATCACAGTAGTTGGCAGCG
Bmp6 R	CCATCACAGTAGTTGGCAGCG
Cp F	AGGTCGCTCCTCACAGCA
Cp R	TGGGGACAGTCCATTCGTA
Dmt1 +IRE F	AGCTAGGGCATGTGGCACTCT
Dmt1 +IRE R	ATGTTGCCACCGCTGGTATC
Dmt1 noIRE F	AGCCCAGCCAGAGCCAAGTA
Dmt1 noIRE R	CCCCCTTTGTAGATGTCCAC
F4/80 (*Adgre1*) F	GGAGGACTTCTCCAAGCCTATT
F4/80 (*Adgre1*) R	AGGCCTCTCAGACTTCTGCTT
Fpn1 F	TGTCAGCCTGCTGTTTGCAGGA
Fpn1 R	TCTTGCAGCAACTGTGTCACC
Gapdh F	TGTCCGTCGTGGATCTGAC
Gapdh R	CCTGCTTCACCACCTTCTTG
Hamp1 F	ATACCAATGCAGAAGAGAAGG
Hamp1 R	AACAGATACCACACTGGGAA
Id1 F	ACCCTGAACGGCGAGATCA
Id1 R	TCGTCGGCTGGAACACATG
IL6 F	GCTACCAAACTGGATATAATCAGGA
IL6 R	CCAGGTAGCTATGGTACTCCAGAA
Rplp0 F	AGATTCGGGATATGCTGTTGGC
Rplp0 R	TCGGGTCCTAGACCAGTGTTC
Saa2 F	AGTCTGCCATGGAGGGTTTT
Saa2 R	CCCGAGCATGGAAGTATTTG
Smad6 F	GTTGCAACCCCTACCACTTC
Smad6 R	GGAGGAGACAGCCGAGAATA
Smad7 F	GCAGGCTGTCCAGATGCTGT
Smad7 R	GATCCCCAGGCTCCAGAAGA
Tf F	GACTCCGAACAACCTGAAGC
Tf R	GCGTAGTAGTAGGTCTGTGGATGTT
Tfr1 F	CCCATGACGTTGAATTGAACCT
Tfr1 R	GTAGTCTCCACGAGCGGAATA
Tfr2 F	GGAGGTCAATTCCCATACCCT
Tfr2 R	CGACCACCAACACGGAGTC
Zip14 F	TGGAACCCTCTACTCCAACG
Zip14 R	CTGAGGGTTGAAGCCAAAAG
